# Competition and feeding ecology in two sympatric *Xenopus* species (Anura: Pipidae)

**DOI:** 10.7717/peerj.3130

**Published:** 2017-04-19

**Authors:** Solveig Vogt, F. André de Villiers, Flora Ihlow, Dennis Rödder, John Measey

**Affiliations:** 1 Centre for Invasion Biology, Department of Botany & Zoology, Stellenbosch University, Stellenbosch, South Africa; 2 Herpetology Section, Zoologisches Forschungsmuseum Alexander Koenig (ZFMK), Bonn, Germany

**Keywords:** Diet, Cannibalism, Alien species, Threatened species, Foraging, Interspecific competition, Trophic niche, Invasive species

## Abstract

The widespread African clawed frog (*Xenopus laevis*) occurs in sympatry with the IUCN Endangered Cape platanna (*Xenopus gilli*) throughout its entire range in the south-western Cape, South Africa. In order to investigate aspects of the interspecific competition between populations of *X. laevis* and *X. gilli*, an assessment of their niche differentiation was conducted through a comprehensive study on food composition and trophic niche structure at two study sites: the Cape of Good Hope (CoGH) and Kleinmond. A total of 399 stomach contents of *X. laevis* (*n* = 183) and *X. gilli* (*n* = 216) were obtained together with samples of available prey to determine food preferences using the Electivity index (E*), the Simpson’s index of diversity (1 − *D*), the Shannon index (*H*′), and the Pianka index (*O*_*jk*_). *Xenopus gilli* diet was more diverse than *X. laevis*, particularly in Kleimond where the Shannon index was nearly double. Both species were found to consume large amounts of tadpoles belonging to different amphibian species, including congeners, with an overall higher incidence of anurophagy than previously recorded. However, *X. laevis* also feeds on adult *X. gilli*, thus representing a direct threat for the latter. While trophic niche overlap was 0.5 for the CoGH, it was almost 1 in Kleinmond, suggesting both species utilise highly congruent trophic niches. Further, subdividing the dataset into three size classes revealed overlap to be higher in small frogs in both study sites. Our study underlines the importance of actively controlling *X. laevis* at sites with *X. gilli* in order to limit competition and predation, which is vital for conservation of the south-western Cape endemic.

## Introduction

Diet and nutrition are widely recognised to represent crucial parameters for understanding life history, population fluctuation, as well as the impact of community modifications (Anderson, Haukos & Anderson, 1999; Dietl, Engels & Solé, 2009). The investigation of species’ feeding ecology yields important insights into nutritional requirements as well as into niche segregation in sympatric species (Guidali, Scali & Carettoni, 2000; Leibold & McPeek, 2006; Knickle & Rose, 2014). If two species overlap in time and space as well the resources they utilise, and one or more of those resources is limiting, interspecific competition occurs (Begon, Townsend & Harper, 2006; Greenlees et al., 2007). Competition for resources in closely related species has been identified as a driver for speciation and niche segregation (Holt, 1977) which makes the assessment of their feeding ecology a powerful tool to explain interspecific competition (Amundsen et al., 2004). Additionally, interspecific competition between invasives that moved into the ranges of closely related native species is widely accepted to negatively affect populations of the latter (Blackburn et al., 2014).

Among amphibians, invasive populations of the African clawed frog, *Xenopus laevis* (Daudin 1802), have one of the highest recorded impacts of all invasive amphibians (Measey et al., 2016; Kumschick et al., 2017). While the species was originally distributed for pregnancy testing and laboratory use (Gurdon & Hopwood, 2000; Van Sittert & Measey, 2016), today large numbers are exported as pets (Herrel & Van der Meijden, 2014; Measey, in Press). Consequently, invasive populations have established on four continents (Measey et al., 2012 and references therein). While some studies exist on invasive populations (McCoid & Fritts, 1989; Measey, 2001; Lobos & Measey, 2002; Rebelo et al., 2010; Measey, 2016), the autecology of *X. laevis* in its native range in southern Africa is poorly studied and confined to few investigations of diet of populations inhabiting artificial water bodies (e.g., Schoonbee, Prinsloo & Nxiweni, 1992).

Frogs of the family Pipidae are unique among anurans in that they lack a tongue (Ridewood, 1897), whereas feeding modes in all other frogs are usually defined by the pattern of tongue protraction. Pipids use a unique set of prey capture modes including inertial suction, jaw prehension, forearm scooping, overhead kicks and terrestrial lunges (Avila & Frye, 1978; Measey, 1998a; O’Reilly, Deban & Nishikawa, 2002; Carreño & Nishikawa, 2010). Within the genus *Xenopus*, these feeding modes have been attributed to the capture of different categories of prey, from benthic, planktonic, carrion, nektonic and terrestrial environments (Measey, 1998b; Lobos & Measey, 2002; Bolnick et al., 2003; Amaral & Rebelo, 2012). Despite the potential to consume diverse prey types, including carrion and other frogs (Measey et al., 2015), most studies have revealed zooplankton and benthic invertebrates to constitute the major components of their diet (Kazadi, Bruyn & Hulselmans, 1986; Schoonbee, Prinsloo & Nxiweni, 1992; De Bruyn, Kazadi & Hulselmans, 1996; Bwong & Measey, 2010).

*Xenopus laevis* is a large pipid (∼130 mm SVL in females), and can occur at extremely high densities (>6 per m^2^; Measey, 2001) causing food resources to become limited (Measey, 1998b). The species has a wide distribution encompassing most of southern Africa, and extending as far north as Malawi (Furman et al., 2015). In contrast, the closely related Cape platanna *Xenopus gilli* Rose and Hewitt, 1927 is much smaller, reaching ∼60 mm SVL in females. *Xenopus gilli* is endemic to the south-western Cape of South Africa, and its range is entirely subsumed by *X. laevis* (Picker & De Villiers, 1989; De Villiers, 2004; Fogell, Tolley & Measey, 2013). While *X. laevis* cannot strictly be considered an invasive species in the distribution of *X. gilli*, its numbers are believed to be greatly inflated in the region due to habitat change, specifically the construction of permanent freshwater impoundments (Picker & De Villiers, 1989; De Villiers, De Kock & Measey, 2016), and it has been termed a ‘domestic exotic’ (Measey et al., 2017). Populations of *X. laevis* have been reported to negatively affect native amphibian communities (Amaral & Rebelo, 2012; Lillo, Faraone & Valvo, 2010; Measey & De Villiers, 2014), with the suggestion that pipids may have a greater proportion of frogs in their diet than all other frogs (Measey et al., 2015). However, its interactions with *X. gilli* in their original habitat remain unclear.

Since its description, there have been concerns about the conservation of *X. gilli*, concentrating on gene introgression through hybridisation with *X. laevis* (Kobel, Pasquier & Tinsley, 1981; Picker, 1985). However, the impact of introgression has been questioned (Evans et al., 1998), and besides habitat change, the greatest threats to *X. gilli* are thought to stem from competition with invading populations of *X. laevis* (Measey, 2011). Several *Xenopus* species are renowned for their cannibalistic tendencies (Measey et al., 2015), and it has been suggested that *X. laevis* can impact populations of *X. gilli* through predation on eggs and tadpoles (Measey, 2011). Picker & De Villiers (1989) suggested that *X. laevis* had competitively excluded *X. gilli* throughout wetland habitats on the Cape Flats. Further evidence that these two *Xenopus* species directly compete comes from the results of removing *X. laevis* in a control programme at the Cape of Good Hope Nature Reserve (CoGH: Picker & De Villiers, 1989). De Villiers, De Kock & Measey (2016) showed that the population of *X. gilli* at CoGH had higher recruitment than those in Kleinmond where *X. laevis* and *X. gilli* occur together at high densities.

In order to investigate the nature of competition between *X. laevis* and sympatric populations of *X. gilli*, we assessed the diet of both species where they occur in sympatry. Niche overlap of the two species was assessed through analyses of prey availability, and the subsequent comparison to stomach contents of adult *X. laevis* and *X. gilli* from two study sites to determine prey selectivity. As predator–prey relations in freshwater environments are particularly size-dependent (Brose et al., 2006), we considered predator size classes within each prey species separately in order to remove the potential for bias from the larger *X. laevis*. Lastly, we assess anurophagy and cannibalism in these natural populations of *Xenopus*.

## Methods

Field research was conducted between July and September 2014 at two study sites, namely, the Cape of Good Hope section of the Table Mountain Nature Reserve (hereafter CoGH) and private land in the vicinity of Kleinmond (hereafter Kleinmond). At both sites, both *Xenopus* species occur sympatrically (Picker & De Villiers, 1989; Evans et al., 1998; Fogell, Tolley & Measey, 2013). At the time of study, the areas were under different management regimes: *X. laevis* were removed annually from CoGH while at Kleinmond they were left (De Villiers, De Kock & Measey, 2016). Both sites consist of a mosaic of permanent impoundments and areas that flood during the austral winter rains (see Table 1). All ponds were visited three days in a row at either two-, or three-week intervals (De Villiers, De Kock & Measey, 2016). Frogs were caught using funnel traps baited with chicken liver contained within a mesh bag to prevent ingestion, set at sunset, and removed within two hours of dawn the following day (approximately 12 h: Measey, 1998b). The majority of dietary samples were obtained by stomach flushing following Measey (1998b). Stomach flushing is a non-lethal method commonly applied to amphibians (Patto, 1998; Solé et al., 2005), and no deleterious effects were observed in either species in response to the procedure. Only stomach content samples from *X. laevis* removed from the CoGH were obtained by dissection in the laboratory (De Villiers, de Kock & Measey, 2016). All other frogs were released at the site of capture immediately after data collection.

**Table 1:** Locations and sizes of examined water bodies in both study sites in the Western Cape, South Africa.

Site	ID	Coordinates (WGS 1984)	Size (m^2^)
CoGH	PP1	34°18′21.0″S, 18°26′27.4″E	757
	PP2	34°18′03.8″S, 18°26′30.1″E	946
	PP3	34°18′47.5″S, 18°26′02.7″E	603
	TP4	34°18′43.6″S, 18°25′48.1″E	39
	TP5	34°18′15.1″S, 18°26′27.0″E	48
Kleinmond	TP6	34°20′02.4″S, 19°05′16.3″E	868
	TP7	34°19′48.6″S, 19°04′56.1″E	1,514
	TP8	34°20′00.1″S, 19°05′02.4″E	2,280

**Notes.**

PP permanent ponds TP temporary ponds

Dietary samples were preserved in 70% ethanol for later examination in the laboratory, where prey items were counted with taxonomic identification to Order level, or lower where possible. It is possible that some prey items flushed from stomachs were ingested within the traps. Therefore, the prey items noted to be attracted to baited traps (i.e., non-*Xenopus* tadpoles and adult pipid frogs), were examined carefully for signs of digestion before inclusion in totals. Ethics approval was granted by Stellenbosch University’s Research Ethics Committee: Animal Care and Use (SU-ACUD15-00011). Permission to capture frogs came from CapeNature (AAA007-01867) and South African National Parks (SANParks CRC/2014-2015/001–2009/V1).

In order to assess prey availability, semi-quantitative sampling of potential prey items from the benthos, nekton and zooplankton was conducted at all ponds studied. Samples of the benthic community were collected using a core-tube-sampler (100 cm ×7 cm), and sieved on location through a 2.5 mm mesh. Nektonic organisms were collected through repeated 2 m sweeps using a handheld dip net (2.5 mm mesh), and zooplankton samples were filtered from randomly selected pond water samples (25 l) using a sieve with 0.3 mm mesh. From each pond, we pooled ten core samples, 25 sweeps and three pond water samples to ensure comparative data on prey availability. Samples were subsequently preserved in 70% ethanol for later examination in the laboratory, where prey items were assigned to habitat classes (benthos, nekton, zooplankton and terrestrial), enumerated (N total number of individuals obtained) and their frequency in frogs’ stomachs (Freq total number of frogs containing that prey item) with taxonomic identification to Order level, or below. Percentages were calculated on the count for individual taxon compared to the sum for all taxa in that class. The volume of prey items was estimated from linear measures (made using a dissecting microscope and digital callipers to the nearest 0.01 mm) using formulae for geometric shapes (ellipsoid) following Colli & Zamboni (1999).

### Data analyses

Studies comparing diversity indices suggest that while common diversity indices appear interchangeable, using several indices provides greater insight into system interactions (Morris et al., 2014). Simpson’s index of diversity (1 − *D*) (Simpson, 1949: equation 1) performs best when differentiating between sites; compound diversity measures discriminate because differences are often based on changes in abundant species (Morris et al., 2014); where *p* is the proportional abundance of resource *i*.

(1) \begin{eqnarray*}1-D= \frac{1}{\sum p_{i}^{2}} .\end{eqnarray*}

Simpson’s index of diversity ranges from 0 (no diversity) to 1 (high diversity), and was used to measure the diversity of prey items available at different sites. Shannon’s diversity (H’) is the best index to describe relationships between organisms, such as predator prey relationships (Morris et al., 2014); where *p* is the proportional abundance of resource *i*.

(2) \begin{eqnarray*}{H}^{{^{\prime}}}=-\sum p_{i}^{2}.\end{eqnarray*}

In order to determine whether the larger *X. laevis* suppresses the smaller *X. gilli* through interspecific competition for food we quantified the overlap in diet between the sympatric populations using the MacArthur & Levins’ index (*O*_*jk*_) (MacArthur & Levins, 1967), as modified by Pianka (1973; equation 3) calculated using the pgirmess package (Giraudoux, 2016) for Cran R 3.1.2 (R Core Team, 2015)

(3) \begin{eqnarray*}{O}_{jk}={O}_{kj}= \frac{\sum _{i}^{n}{p}_{ij}\times {p}_{ik}}{\sqrt{\sum _{i}^{n}{p}_{ij}^{2}}} \frac{\sum _{i}^{n}{p}_{ij}\times {p}_{ik}}{\sqrt{\sum _{i}^{n}{p}_{ij}^{2}\times \sum _{i}^{n}{p}_{ik}^{2}}} \end{eqnarray*}

where *P*_*ij*_ and *P*_*ik*_ are the proportions of the *i*th resource used by the *j*th and the *k*th species respectively and *n* is the number of resource categories. *O*_*jk*_ determines dietary overlap between the species pair as ranging from 0 (no overlap) to 1 (complete overlap). Significance of *O*_*jk*_ was assessed using a null-model computed with the niche_null_model function of the EcoSim package (Gotelli, Hart & Ellison, 2015) for Cran R. Confidence Intervals calculated refer to the null model (rather than the index) in those cases where the observed *O*_*jk*_ is outside of this distribution and the overlap is statistically significant. The same indices were calculated for available prey sampled in the environment (see above). For these measures, all samples were pooled for each site: CoGH and Kleinmond. Food preferences of both *Xenopus* species were assessed using the Electivity index (E*) (Jacobs, 1974: equation 4)

(4) \begin{eqnarray*}{E}_{i}^{\ast }= \frac{{r}_{i}-{p}_{i}}{{r}_{i}+{p}_{i}-2{r}_{i}{p}_{i}} \end{eqnarray*}

based on the proportions of food category *i* in the diet (*r*_*i*_) and in the environment (*p*_*i*_), which determines electivity ranging from −1, which indicates total avoidance, to 0 indicating use in proportion to availability, to 1, indicating preference. Following Measey (1998b) electivity was not computed for prey items with a total dietary frequency below 10. Significances of electivity were assessed using Chi-square tests followed by building 95% Bonferroni confidence intervals (see Neu, Byers & Peek, 1974; Beyers & Steinhorst, 1984). Significance was determined at α = 0.05.

Predator-prey relations in freshwater environments are highly size-dependent (Brose et al., 2006). Because of the pronounced size disparity between the two species (e.g., Fogell, Tolley & Measey, 2013), we subdivided the analysis on competition into three size classes for both species: two that cover overlapping size ranges for small (30–52 mm SVL) and medium (52–72 mm), and one for the largest *X. laevis* (>72 mm) (see Tables S1–S6) to prevent a potential bias due to the larger body size of *X. laevis*. Measey (1998b) suggested that diet of clawed frogs may be influenced by size and sex, making three factors of interest with our primary interest on the difference between species. All statistical analyses and calculations were conducted with Cran R 3.1.2.

## Results

A total of 399 stomach contents was collected from both sites, CoGH (*n*_*X*.*laevis*_ = 94, *n*_*X*.*gilli*_ = 111) and Kleinmond ( *n*_*X*.*laevis*_ = 89, *n*_*X*.*gilli*_ = 105). Less than 2% of all collected prey items could not be identified, mostly because they were too digested or fragmented to be recognised. We identified 21 taxa from stomach content samples of both *Xenopus* species (*n*_*X*.*laevis*_ = 16, *n*_*X*.*gilli*_ = 19), comprising 12 terrestrial and nine aquatic taxa including eggs, larvae and adult frogs (Tables 2 and 3). In addition to these prey items, stomach contents also contained sloughed skin (16%; *n*_*X*.*laevis*_ = 17, *n*_*X*.*gilli*_ = 49), vegetal matter (14%; *n*_*X*.*laevis*_ = 34, *n*_*X*.*gilli*_ = 23) and stones (∼1%, *n*_*X*.*laevis*_ = 2, *n*_*X*.*gilli*_ = 1).

**Table 2:** Prey categories consumed by *Xenopus laevis*, *Xenopus gilli* and obtained during habitat sampling at the Cape of Good Hope (CoGH). Consumed sloughed skin, plant matter, and stones not shown for clarity. Prey categories with environmental abundances (Ne, Ne% and Ve) of <1% are shown in grey. N is the total number of individuals obtained in all samples; N% is the percentage of N compared with the total individuals in the entire sample; V is the summed volume of individuals; Freq is the number of stomachs found containing this taxon; E* is the Jacobs (1974) Electivity index; χ^2^ = Chi-square residuals, significant values are marked with an asterisk.

	Environment			*Xenopus laevisn* = 94						*Xenopus gillin* = 111					
CoGH	Ne	Ne (%)	Ve	N	N (%)	V	Freq	E*	*χ*^2^	N	N (%)	Freq	V	E*	*χ*^2^
Anisoptera	38	1.32	2656.01	27	2.65	1394.94	14	0.34	0.24	11	0.66	6	343.08	−0.34	−2.35*
Brachycera	0	0.00	0.00	3	0.29	56.93	1	1		0	–	–	–		
Coleoptera	9	0.31	8405.49	20	1.96	199.16	11	0.73	6.62*	30	1.79	22	195.34	0.71	11.73*
**Ephemeroptera**	8	0.28	59.36	0	0.00	0.00	–	−1	−2.37*	3	0.18	3	0.16	−0.22	−0.76
Heteroptera	61	2.12	660.07	15	1.47	152.81	8	−0.18	−4.26*	1	0.06	1	0.70	−0.95	−5.78*
Hymenoptera	0	0.00	0.00	9	0.88	8.15	4	1		3	0.18	3	0.00	1.00	
Nematocera	49	1.71	68.42	65	6.39	19.39	19	0.59	5.54*	23	1.37	15	7.89	−0.11	−0.83
Neuroptera	0	0.00	0.00	1	0.10	0.00	1	1		0	–	–	–		
Psocoptera	1	0.03	0.78	0	0.00	0.00	–	−1	−0.84	0	–	–	–	−1.00	−0.76
Trichoptera	29	1.01	106.48	14	1.38	55.18	7	0.16	−0.53	40	2.39	23	94.52	0.41	6.15*
Zygoptera	2368	82.42	55275.07	24	2.36	390.33	15	−0.99	−0.84	113	6.75	37	1983.17	−0.97	−0.76
Zygentoma	1	0.03	1.41	0	0.00	0.00	–	−1	−40.14*	0	–	–	–	−1.00	−33.94*
**Amphipoda**	7	0.24	14.26	43	4.22	416.45	10	0.90	24.36*	497	29.71	29	1463.16	0.99	245.74*
*Daphnia*	98	3.41	139.67	0	0.00	0.00	–	−1	−8.31*	493	29.47	6	91.84	0.84	57.88*
Ostracoda	173	6.02	26.24	586	57.56	88.87	34	0.91	127.9*	352	21.04	25	53.38	0.61	26.84*
Aranae	1	0.03	1.28	0	0.00	0.00	0	−1	−0.84	0	–	–	–	−1.00	−0.76
Acari	13	0.45	0.42	139	13.65	4.61	28	0.94	57.48*	51	3.05	8	1.37	0.75	16.20*
Scorpiones	0	0.00	0.00	0	0.00	0.00	–	–		0	–	–	–		
Anura	17	0.59	3092.02	68	6.71	12368.1	49	0.71	16.2*	24	1.40	22	4365.2	1.00	4.51*

**Table 3:** Prey categories consumed by *Xenopus laevis*, *Xenopus gilli* and obtained during habitat sampling at Kleinmond. Consumed sloughed skin, plant matter, and stones not shown for clarity. Prey categories with environmental abundances (Ne, Ne% and Ve) of <1% are shown in grey. N is the total number of individuals obtained in all samples; N% is the percentage of N compared with the total individuals in the entire sample; V is the summed volume of individuals; Freq is the number of stomachs found containing this taxon; E* is the Jacobs (1974) Electivity index; χ^2^ = Chi-square residuals, significant values are marked with an asterisk.

Kleinmond	Ne	Ne (%)	Ve	N	N (%)	V	Freq	E*	*χ*^2^	N	N (%)	V	Freq	E*	*χ*^2^
Blattodea	0	–	–	0	–	–	–			2	0.22	62.38	1		
Brachycera	0	–	–	1	0.06	0.00	1	1		0	–	–	–		
Coleoptera	260	15.09	4.45	65	3.84	1053.55	33	−0.63	−11.62*	123	13.82	1127.53	41	−0.05	0.08
Collembola	0	–	–	0	–	–	–			1	0.11	0.07	1		
**Ephemeroptera**	9	0.52	0.08	0	–	–	–			0	–	–	–		
Heteroptera	166	9.63	1.16	22	1.30	231.21	11	−0.78	−10.89*	12	1.35	65.84	7	−0.77	−7.64*
Hymenoptera	1	0.06	0.00	0	–	–	–			0	–	–	–		
Nematocera	2	0.12	0.00	9	0.53	5.55	6	0.64	5.06*	14	1.57	113.53	10	0.86	13.23*
Sternorrhyncha	0	–	–	0	–	–	–			1	0.11	0.29	1		
Thysanoptera	0	–	–	0	–	–	–			1	0.11	0.05	1		
Trichoptera	0	–	–	3	0.18	2.08	2			6	0.67	11.29	4		
Zygoptera	0	–	–	1	0.06	24.05	1			0	–	–	–		
Amphipoda	496	28.79	0.93	6	0.35	18.09	6	−0.98	−21.68*	32	3.60	590.05	13	−0.83	−13.45*
*Daphnia*	1	0.06	0.00	440	25.99	1115.18	4	1.00	445.41*	235	26.40	581.89	15	1.00	336.67*
Ostracoda	0	–	–	1	0.06	0.15	1			23	2.58	3.49	10		
Acari	11	0.64	0.03	1	0.06	1.41	1	−0.83	−2.96*	4	0.45	7.54	3	−0.17	−0.58
Aranae	0	–	–	0	–	–	–	−1	−0.99	1	0.11	0.43	1	1	−0.70
Pseudoscorpiones	1	0.06	0.00	0	–	–	–			–	–	–	–		
Annelida	0	–	–	0	–	–	–			1	0.11	485.36	1		
Anura	776	45.04	93.35	1131	69.32	12692.76	66	1	13.59*	412	47.47	9697.35	63	1	1.83*

### Availability of prey items

Simpson’s index of diversity (1 − *D*) shows that the diversity of prey items available was more than twice has high in CoGH than in Kleinmond (CoGH: 1 − *D* = 0.68; Kleinmond: 1 − *D* = 0.28). In the CoGH, by far the most abundant available prey items were zygopterans representing >80% and ostracods representing 6% while all other classes contributed less than 5%. In Kleinmond, anurans (45%), amphipods (29%) and coleopterans (15%) represented the most abundant prey item classes (Tables 2 and 3). Aquatic prey appeared in abundance at both sites, with more, smaller prey at the CoGH (mean volume: 24.5 mm^3^ ± 2.54 SE) and fewer, larger prey in Kleinmond (mean volume: 60.2 mm^3^ ± 7.49 SE) at a ratio of 5:2, respectively.

### Interspecific overlap

Shannon’s diversity (*H*’) suggests that the diversity of prey consumed at CoGH was very similar between species (*H*’ for *X. laevis* 2.31 and 2.55 for *X. gilli*). In Kleinmond, however, *X. gilli* consumed nearly twice the diversity (*H*’ 2.58) of prey items than those consumed by *X. laevis* (*H*’ 1.64). Niche overlap (*O*_*jk*_) between *X. laevis* and *X. gilli* was 0.491 (95% CI [0.550–0.825], p_lower tail_  > 0.999, *p*_upper tail_ < 0.001) in the CoGH and 0.965 (95% CI [0.415–0.785], *p*_lower tail_ > 0.999, *p*_upper tail_ < 0.001) at Kleinmond. When size classes were analysed separately, niche overlap (*O*_*jk*_) in the CoGH was 0.5 (95% CI [0.010–0.677], *p*_lower tail_ > 0.879, *p*_upper tail_ < 0.121) for small and 0.2 (95% CI [0.023–0.840], *p*_lower tail_ >  0.655, *p*_upper tail_ < 0.345) for larger frogs while overlap was almost complete for both size classes (small 0.92: 95% CI [0.009–0.861], *p*_lower tail_ >  0.999, *p*_upper tail_ < 0.001 and large 0.96 95% CI [0.005–0.626], *p*_lower tail_ >  0.997, *p*_upper tail_ < 0.003) in Kleinmond.

### Anurophagy

In terms of prey frequency, anuran larvae and eggs of various species, including *Xenopus*, were found to represent a major component of the diet of *X. laevis* (67% of all prey items) and *X. gilli* (47% of all prey items; Tables 2 and 3). Subdividing anurans into non-*Xenopus* (eggs, tadpole and adults) and *Xenopus* revealed both *X. laevis* and *X. gilli* feed predominantly on tadpoles of non-*Xenopus* species (CoGH: N%_*X. laevis*_ = 3.35, N%_*X.gill*_ = 0.85; Kleinmond: N%_*X. laevis*_ = 2.48, N%_*X. gill*_ = 5.53). Eggs were also consumed (CoGH: N%_*X. laevis*_ = 0.59, N%_*X. gill*_ = 0.37; Kleinmond: N%_*X. laevis*_ = 0.83, N%_*X. gill*_ = 1.50), while consumption of tadpoles and eggs of *Xenopus* (i.e., potential cannibalism) was negligible (Freq < 5). Considering size classes separately revealed small frogs of both species to feed on non-*Xenopus* tadpoles at both study sites (CoGH: N%_*X. laevis*_ = 3.25, N%_*X. gill*_ = 1.00; Kleinmond: N%_*X. laevis*_ = 2.62, N%_*X. gill*_ = 11.01) which was also true for medium sized frogs in Kleinmond (N%_*X. laevis*_ = 1.83, N%_*X. gill*_ = 9.43).

Adult non-*Xenopus* frogs consumed (all *Cacosternum australis*; SVLs 20.5, 22.7 and 21.2 mm) were found in dietary samples of both *Xenopus* species in Kleinmond, but in the CoGH an *X. laevis* (SVL 79.6 mm) was found to prey on adult *X. gilli* (SVL 36.9 mm). Anurophagy differed greatly between Kleinmond, where the ratio between anurans and total prey was 0.47 for *X. gilli* and 0.67 for *X. laevis*, to much lower levels at the CoGH where the same ratio was 0.01 for both *X. gilli* and *X. laevis*.

## Discussion

Previous studies have documented the presence of competition between *Xenopus gilli* and *X. laevis*, evidenced by a reduction in recruitment of *X. gilli* while *X. laevis* increases in abundance (De Villiers, De Kock & Measey, 2016; Picker & De Villiers, 1989). For one aspect of this competition, we show a large dietary niche overlap of ∼50% in the Cape of Good Hope reserve and almost complete overlap (97%) in Kleinmond, suggesting a high level of competition for food resources between the two species. Our analysis of prey volume revealed that the larger *X. laevis* are likely to impact greatly on available food items through predation. This information combined with the knowledge that *X. laevis* typically outnumbers *X. gilli* around 3:1 (De Villiers, De Kock & Measey, 2016) suggests that competition for finite prey resources is likely to be a serious impediment to the survival of *X. gilli*. Also, we also found direct predation of adult *X. gilli* by *X. laevis*, an interaction previously only speculated (Picker & De Villiers, 1989; Fogell, Tolley & Measey, 2013).

Studies on diet of *Xenopus* species suggest that they do not remain static, but adapt together with prey availability throughout the year (see Measey, 1998b). A study of diet during summer of 1983 in the CoGH showed that the prey consumed in these permanent ponds remains very similar (Simmonds, 1985) to the results we show for winter. Interestingly, Simmonds (1985) recorded many *Xenopus* eggs and larvae in the stomachs, but does not mention the high number of tadpoles of other species that we found. Although Simmonds suggests that consumption of tadpoles could be related to them being confined in traps, we found that many of those we removed from stomachs were partially digested, suggesting ingestion prior to entering traps. Measey et al. (2015) calculated the proportion of amphibian prey from 355 records of 228 species of anurans, finding that pipids have (on average) the highest proportion of anurans in their diet, while the highest proportion previously recorded in a single study was in *Lithobates catesbeiana* which had an anurophagy proportion of 0.19 (Leivas, Leivas & Moura, 2012). In this study, *X. laevis* and *X. gilli* in Kleinmond were found to have an anurophagy proportion of 0.67 and 0.47, although these proportions were much lower at CoGH (0.01 for both species). Our data, therefore, shows that the diet of *X. laevis* from Kleinmond comprises three and a half times the proportion of amphibians than any other known adult anuran, confirming the importance of anurophagy for pipids in general and at this site in particular.

Our study determined some differences in diet between sites. At the CoGH, *X. gilli* preys on a large variety of different prey taxa, utilising a wider and more diverse niche than in Kleinmond. While the niche of *X. laevis* was broader at the CoGH it was more diverse in Kleinmond where availability of potential prey items was mainly restricted to anuran eggs and larvae. In addition, consumption of terrestrial prey items was significantly higher in both species in Kleinmond suggesting that the restricted diversity of available aquatic prey induces *Xenopus* to catch terrestrial prey as reported by Measey (1998b). The same author also suggested terrestrial prey might represent an important component of the diet of *X. laevis*, and this might particularly apply to sites with a restricted aquatic food supply. Amounts of terrestrial prey were higher in *X. laevis* than in *X. gilli*, but compared to prevalence of aquatic prey, low at both sites. Aquatic prey was apparently in abundance at both sites, with very few animals having empty stomachs.

Our data suggest that dietary competition is not equal among size classes with increased competition between smaller individuals. This is of note as the larger *X. laevis* is likely to grow faster (see McCoid & Fritts, 1989; Measey, 2001) and be under this more intense competition for a shorter period of their lives. While our study reveals from a single sampling point how dietary resources are partitioned between these species, competition occurs over the life of individuals. With abundant prey, we show that sympatric *Xenopus* species do have a large dietary overlap, but direct competition for dietary resources may only occur when these resources are limited. Presumably, the ongoing removal of *X. laevis* from the CoGH keeps competition there at a very low level. However, in Kleinmond, not only do *X. laevis* outnumber *X. gilli* at a ratio of 3:1 (De Villiers, De Kock & Measey, 2016), but sites dry annually which may provoke increased competition as water levels fall. In addition, we do not consider here the competition between larvae, or for other limited resources such as egg deposition locations at either site, although these would be important over the life of individuals.

Food composition observed for *X. laevis* is generally in accordance with earlier studies (Schoonbee, Prinsloo & Nxiweni, 1992; Measey, 1998b; Lobos & Measey, 2002; Faraone et al., 2008; Lillo, Faraone & Valvo, 2010; Amaral & Rebelo, 2012). While *X. laevis* has previously been reported to negatively affect native amphibian populations (Crayon, 2005; Rebelo et al., 2010; Lillo, Faraone & Valvo, 2010; Amaral & Rebelo, 2012), by consuming tadpoles and eggs (Schoonbee, Prinsloo & Nxiweni, 1992; Faraone et al., 2008), here we report them to prey on adult frogs, including its endangered conspecific *X. gilli*. Thus, *X. laevis* is a direct predator of *X. gilli*. In this study, the other native amphibians consumed included tadpoles of the common Cape River Frog *Amietia fuscigula* and adults and tadpoles of the southern dainty frog *Cacosternum australis*. Measey & De Villiers (2014) previously reported consumption of the clicking stream frog *Strongylopus grayii* at the same site near Kleinmond.

Dietary samples also contained sloughed skin, plant matter and stones, also reported by Measey (1998b), Faraone et al. (2008) and Amaral & Rebelo (2012). However, pipid frogs are known for their inertial suction feeding method (Sokol, 1969) which likely leads to the accidental ingestion of soil or plant matter. While previous research from South Wales and Sicily (Measey, 1998b; Faraone et al., 2008) found in the diet of invasive *X. laevis* that zooplanktonic components represent the numerically most abundant prey group, our results partly support this result for both species in the CoGH but suggest that *Xenopus* mainly consume nektonic prey (in terms of volume and frequency). However, benthic organisms represented the numerically most abundant prey for both populations of *X. laevis* from Chile (Lobos & Measey, 2002).

Neither *Xenopus* species was found to take prey in the same proportion as it occurred in the environment. The low consumption of some abundant prey taxa at each site (e.g., Zygoptera at the CoGH or Amphipoda in Kleinmond) combined with a selection for other taxa (e.g., *Daphnia*, amphibian larvae and eggs) indicates that resource use was not random and not exclusively determined by availability, agreeing with previous assessments (Measey, 1998b). Thus, both species seem to select similar resources from within the environment. According to MacArthur & Pianka (1966), optimal foragers are typically expected to choose prey according to profitability irrespective of density. However, preferences of both species were not entirely consistent across sampling localities. Handling time for different prey items, especially for predators such as *Xenopus*, which are capable of many different feeding modes, is likely to vary widely. The preference that we observe for zooplankters may represent the very small handling time involved in suction feeding compared to actively swimming and/or lunging after nektonic prey. Ultimately, prey choice may result from a great many factors including individual variation in diet, which has been found in a number of amphibian, fish and some avian species (Bolnick et al., 2002; Araújo et al., 2008; Thiemann et al., 2011; Schriever & Williams, 2013). This variation is not simply due to different choices of prey taxa, but rather because some animals exhibit very specialised diets, while other individuals are more generalist.

Interspecific competition is an important factor in the structuring of predatory communities (Caro & Stoner, 2003), usually involving a dominant and an inferior competitor (Holt, 1977; Rehage, Barnett & Sih, 2005; Harrington et al., 2009). In some competitive interactions, even direct aggression is involved (Hersteinsson & Macdonald, 1992; Harrington et al., 2009), leading to the death of the inferior competitor (Palomares & Caro, 1999) or resulting in mutual consumption. Our results agree with the previously demonstrated dominant position of *X. laevis* in the competition with *X. gilli* (De Villiers, De Kock & Measey, 2016); through increased resource use by larger individuals, and direct predation on *X. gilli* eggs, larvae and adults. Therefore, this study supports the continued removal of *X. laevis* in the CoGH. The conservation of *X. gilli* in Kleinmond and at other sites will rely on new plans to remove its congeneric competitor, *X. laevis*.

##  Supplemental Information

10.7717/peerj.3130/supp-1 Data S1 Diet and potential prey data for *Xenopus gilli* and *Xenopus laevis* in Cape of Good Hope and Kleinmond This file contains the raw data for all items stomach flushed from *Xenopus gilli* and *Xenopus laevis* in Cape of Good Hope and Kleinmond, as well as data on potential prey from sampling of their aquatic environments. Each prey item has a single row with the sampling date, sampling method, site name and identification of the item to Order level. Where appropriate the identification, size and sex of the predator is recorded.

10.7717/peerj.3130/supp-2 File S1 Supplementary Tables for prey of *Xenopus* species Prey categories consumed by *Xenopus laevis*, *Xenopus gilli* and obtained during habitat sampling at the Cape of Good Hope and Kleinmond for individuals divided into size categories (small, medium and large).

## Additional Information and Declarations

### Field Study Permissions

The following information was supplied relating to field study approvals (i.e., approving body and any reference numbers): CapeNature (AAA007-01867) and South African National Parks (SANParks CRC/2014-2015/001–2009/V1).

### Data Availability

The following information was supplied regarding data availability: The raw data has been supplied as a Supplementary File.
