# Supplementary material for: Competition and feeding ecology in two sympatric Xenopus species (Anura: Pipidae)
Source: PeerJ. 2017 Apr 19;5:e3130. doi: 10.7717/peerj.3130 (PMC5399871; doi:10.7717/peerj.3130)
Supplement: File S1 — Prey categories consumed by Xenopus laevis, Xenopus gilli and obtained during habitat sampling at the Cape of Good Hope and Kleinmond for individuals divided into size categories (small, medium and large). [file peerj-05-3130-s002.docx]

**Supplemental Files**

**Table S1:** Prey categories consumed by *Xenopus laevis*, *Xenopus gilli* and obtained during habitat sampling at the CoGH for small frogs.

| **CoGH**  **30 – 52 mm** | **Environment** | | ***Xenopus laevis*** n = 74 | | | | |  | ***Xenopus gilli*** n = 36 | | | | |  |
| --- | --- | --- | --- | --- | --- | --- | --- | --- | --- | --- | --- | --- | --- | --- |
| **Order** | Ne | Ne (%) | N | N (%) | Freq | *IRI* (%) | *E** | *χ^2^* | N | N (%) | Freq | *IRI* (%) | *E** | *χ^2^* |
| Anisoptera | 38 | 1.32 | 23 | 2.58 | 12 | 35.76 | 0.35 | 3.561 | 10 | 0.67 | 5 | 2.91 | -0.32 | -2.105 |
| Coleoptera | 9 | 0.31 | 11 | 1.23 | 7 | 2.61 | 0.62 | 5.154 | 23 | 1.54 | 17 | 7.60 | 0.67 | 8.643* |
| Ephemeroptera | 8 | 0.28 | 0 | 0 | 0 | 0.00 | -1.00 | -1.530* | 3 | 0.2 | 3 | 0.04 | -0.15 | -0.521 |
| Heteroptera | 61 | 2.12 | 6 | 0.67 | 4 | 0.85 | -0.50 | -2.806* | 0 | 0 | 0 | 0.00 | -1.00 | -5.556* |
| Hymenoptera | 0 | 0 | 0 | 0 | 0 | 0.00 |  |  | 1 | 0.07 | 1 | 0.00 | NA | NA |
| Nematocera | 49 | 1.71 | 37 | 4.15 | 17 | 3.50 | 0.45 | 5.982* | 22 | 1.47 | 14 | 1.59 | -0.06 | -0.562 |
| Neuroptera | 0 | 0 | 1 | 0.11 | 1 | 0.01 |  |  | 0 | 0 | 0 | 0.00 | NA | NA |
| Psocoptera | 1 | 0.03 | 0 | 0 | 0 | 0.00 | -1.00 | -0.541* | 0 | 0 | 0 | 0.00 | -1.00 | -0.711* |
| Trichoptera | 29 | 1.01 | 3 | 0.34 | 3 | 0.19 | -0.48 | -1.884* | 36 | 2.41 | 20 | 6.07 | 0.43 | 5.566* |
| Zygentoma | 1 | 0.03 | 0 | 0 | 0 | 0.00 | -1.00 | -0.541* | 0 | 0 | 0 | 0.00 | -1.00 | -0.711* |
| Zygoptera | 2368 | 82.42 | 14 | 1.57 | 9 | 6.07 | -0.99 | -25.796* | 89 | 5.95 | 28 | 91.36 | -0.97 | -32.047* |
| Amphipoda | 7 | 0.24 | 31 | 3.48 | 5 | 1.30 | 0.88 | 20.225* | 407 | 27.22 | 23 | 100.00 | 0.99 | 214.355* |
| *Daphnia* | 98 | 3.41 | 0 | 0 | 0 | 0.00 | -1.00 | -5.356* | 477 | 31.91 | 5 | 11.51 | 0.87 | 60.689* |
| Ostracoda | 173 | 6.02 | 578 | 64.87 | 33 | 100.00 | 0.94 | 74.106* | 336 | 22.47 | 20 | 31.99 | 0.65 | 26.552* |
| Acari | 13 | 0.45 | 138 | 15.49 | 27 | 18.54 | 0.95 | 68.791* | 51 | 3.41 | 8 | 1.85 | 0.78 | 17.318* |
| Aranae | 1 | 0.03 | 0 | 0 | 0 | 0.00 | -1.00 | -0.541* | 0 | 0 | 0 | 0.00 | -1.00 | -0.711* |
| Anuran egg | 0 | 0 | 0 | 0 | 0 | 0.00 | 0.35 | 3.561* | 1 | 0.07 | 1 | 0.01 | NA | NA |
| Tadpole spec | 0 | 0 | 29 | 3.25 | 26 | 3.69 | 0.62 | 5.154* | 15 | 1 | 14 | 0.94 | NA | NA |
| *Xenopus* tadpole | 17 | 0.59 | 0 | 0 | 0 | 0.00 | -1.00 | -1.530* | 0 | 0 | 0 | 0.00 | -1.00 | -2.933* |
| N, total number of individuals obtained; N% percentage of N; Freq, frequency of occurrence; Freq % percentages of frequency of occurrence; *IRI* (%), index of relative importance; *E**, Electivity index; *χ^2^* = Chi-square residuals, significant values are marked with an asterisk. | | | | | | | | | | | | | | |

**Table S2:** Prey categories consumed by *Xenopus laevis*, *Xenopus gilli* and obtained during habitat sampling at the CoGH for large frogs.

| **CoGH**  **52 – 72 mm** | **Environment** | | ***Xenopus laevis*** n = 12 | | | | |  | ***Xenopus gilli*** n = 26 | | | | |  |
| --- | --- | --- | --- | --- | --- | --- | --- | --- | --- | --- | --- | --- | --- | --- |
| **Order** | Ne | Ne (%) | N | N (%) | Freq | *IRI* (%) | *E** | *χ^2^* | N | N (%) | Freq | *IRI* (%) | *E** | *χ^2^* |
| Anisoptera | 38 | 1.32 | 0 | 0 | 0 | 0.00 | -1.00 | -0.891* | 1 | 0.56 | 1 | 1.22 | -0.36 | -0.767 |
| Coleoptera | 9 | 0.31 | 9 | 12.86 | 4 | 91.12 | 0.97 | 20.326* | 7 | 3.93 | 5 | 3.77 | 0.87 | 9.179 |
| Ephemeroptera | 8 | 0.28 | 0 | 0 | 0 | 0.00 | -1.00 | -0.409* | 0 | 0 | 0 | 0.00 | -1.00 | -0.667* |
| Heteroptera | 61 | 2.12 | 8 | 11.43 | 3 | 73.39 | 0.75 | 5.959 | 1 | 0.56 | 1 | 0.09 | -0.55 | -1.301 |
| Hymenoptera | 0 | 0 | 1 | 1.43 | 1 | 1.82 | NA | NA | 2 | 1.12 | 2 | 0.30 | NA | NA |
| Nematocera | 49 | 1.71 | 27 | 38.57 | 1 | 33.25 | 0.96 | 25.679* | 1 | 0.56 | 1 | 0.08 | -0.47 | -1.047 |
| Neuroptera | 0 | 0 | 0 | 0 | 0 | 0.00 | NA | NA | 0 | 0 | 0 | 0.00 | NA | NA |
| Psocoptera | 1 | 0.03 | 0 | 0 | 0 | 0.00 | -1.00 | -0.145* | 0 | 0 | 0 | 0.00 | -1.00 | -0.236* |
| Trichoptera | 29 | 1.01 | 4 | 5.71 | 1 | 8.59 | 0.75 | 4.362 | 4 | 2.25 | 3 | 2.05 | 0.43 | 1.877 |
| Zygentoma | 1 | 0.03 | 0 | 0 | 0 | 0.00 | -1.00 | -0.145* | 0 | 0 | 0 | 0.00 | -1.00 | -0.236* |
| Zygoptera | 2368 | 82.42 | 8 | 11.43 | 4 | 100.00 | -0.94 | -5.89*5 | 24 | 13.48 | 9 | 100.00 | -0.93 | -9.394* |
| Amphipoda | 7 | 0.24 | 3 | 4.29 | 2 | 71.84 | 0.91 | 7.464 | 90 | 50.56 | 6 | 54.42 | 1.00 | 143.521* |
| *Daphnia* | 98 | 3.41 | 0 | 0 | 0 | 0.00 | -1.00 | -1.431* | 16 | 8.99 | 1 | 1.27 | 0.52 | 4.513 |
| Ostracoda | 173 | 6.02 | 0 | 0 | 0 | 0.00 | -1.00 | -1.901* | 16 | 8.99 | 5 | 6.30 | 0.27 | 2.051 |
| Acari | 13 | 0.45 | 1 | 1.43 | 1 | 1.17 | 0.58 | 1.398 | 0 | 0 | 0 | 0.00 | -1.00 | -0.851* |
| Aranae | 1 | 0.03 | 0 | 0 | 0 | 0.00 | -1.00 | -0.145* | 0 | 0 | 0 | 0.00 | -1.00 | -0.236* |
| Anuran egg | 0 | 0 | 6 | 8.57 | 6 | 42.05 | NA | NA | 5 | 2.81 | 5 | 1.89 | NA | NA |
| Tadpole spec | 0 | 0 | 1 | 1.43 | 1 | 1.17 | NA | NA | 0 | 0 | 0 | 0.00 | NA | NA |
| *Xenopus* tadpole | 17 | 0.59 | 0 | 0 | 0 | 0.00 | -1.00 | -0.596* | 0 | 0 | 0 | 0.00 | -1.00 | -0.973* |
| N, total number of individuals obtained; N% percentage of N; Freq, frequency of occurrence; Freq % percentages of frequency of occurrence; *IRI* (%), index of relative importance; *E**, Electivity index; *χ^2^* = Chi-square residuals, significant values are marked with an asterisk. | | | | | | | | | | | | | | |

**Table S3:** Prey categories consumed by *Xenopus laevis* and obtained during habitat sampling at the CoGH for very large frogs.

| **CoGH**  **>72 mm** | **Environment** | | ***Xenopus laevis*** n = 10 | | | | |  |
| --- | --- | --- | --- | --- | --- | --- | --- | --- |
| **Order** | Ne | Ne (%) | N | N (%) | Freq | *IRI* (%) | *E** | *χ^2^* |
| Anisoptera | 38 | 1.32 | 4 | 7.02 | 2 | 37.30 | 0.83 | 5.487 |
| Brachycera | 0 | 0 | 3 | 5.26 | 1 | 9.45 | NA | NA |
| Ephemeroptera | 8 | 0.28 | 0 | 0 | 0 | 0.00 | -1.00 | -0.299* |
| Heteroptera | 61 | 2.12 | 1 | 1.75 | 1 | 0.95 | 0.19 | 0.386 |
| Hymenoptera | 0 | 0 | 8 | 14.04 | 3 | 24.60 | NA | NA |
| Nematocera | 49 | 1.71 | 1 | 1.75 | 1 | 0.99 | 0.30 | 0.612 |
| Psocoptera | 1 | 0.03 | 0 | 0 | 0 | 0.00 | -1.00 | -0.106* |
| Trichoptera | 29 | 1.01 | 7 | 12.28 | 3 | 25.23 | 0.93 | 11.728 |
| Zygentoma | 1 | 0.03 | 0 | 0 | 0 | 0.00 | -1.00 | -0.106* |
| Zygoptera | 2368 | 82.42 | 2 | 3.51 | 2 | 14.95 | -0.97 | -4.755* |
| Amphipoda | 7 | 0.24 | 9 | 15.79 | 3 | 100.00 | 0.99 | 31.902* |
| *Daphnia* | 98 | 3.41 | 0 | 0 | 0 | 0.00 | -1.00 | -1.046* |
| *Ostracoda* | 173 | 6.02 | 8 | 14.04 | 1 | 7.76 | 0.68 | 4.364 |
| Acari | 13 | 0.45 | 0 | 0 | 0 | 0.00 | -1.00 | -0.381* |
| Aranae | 1 | 0.03 | 0 | 0 | 0 | 0.00 | -1.00 | -0.106* |
| Tadpole spec | 0 | 0 | 10 | 17.54 | 6 | 57.13 | NA | NA |
| *Xenopus* tadpole | 17 | 0.59 | 0 | 0 | 0 | 0.00 | -1.00 | -0.436* |
| N, total number of individuals obtained; N% percentage of N; Freq, frequency of occurrence; Freq % percentages of frequency of occurrence; *IRI* (%), index of relative importance; *E**, Electivity index; *χ^2^* = Chi-square residuals, significant values are marked with an asterisk. | | | | | | | | |

**Table S4:** Prey categories consumed by *Xenopus laevis*, *Xenopus gilli* and obtained during habitat sampling at the Kleinmond for small frogs.

| **Kleinmond**  **30 – 52 mm** | **Environment** | | ***Xenopus laevis*** n = 15 | | | | |  | ***Xenopus gilli*** n = 36 | | | | |  |
| --- | --- | --- | --- | --- | --- | --- | --- | --- | --- | --- | --- | --- | --- | --- |
| **Order** | Ne | Ne (%) | N | N (%) | Freq | *IRI* (%) | *E** | *χ^2^* | N | N (%) | Freq | *IRI* (%) | *E** | *χ^2^* |
| Blattodea | 0 | 0 | 0 | 0 | 0 | 0.00 | NA | NA | 2 | 0.52 | 1 | 0.05 | NA | NA |
| Brachycera | 0 | 0 | 1 | 0.92 | 1 | 0.11 | NA | NA | 0 | 0 | 0 | 0.00 | NA | NA |
| Coleoptera | 260 | 15.09 | 10 | 9.17 | 7 | 8.47 | -0.25 | -1.406 | 85 | 22.31 | 23 | 100.00 | 0.32 | 4.960* |
| Collembola | 0 | 0 | 0 | 0 | 0 | 0.00 | NA | NA | 1 | 0.26 | 1 | 0.03 | NA | NA |
| Ephemeroptera | 9 | 0.52 | 0 | 0 | 0 | 0.00 | -1.00 | -0.733* | 0 | 0 | 0 | 0.00 | -1.00 | -1.315* |
| Heteroptera | 166 | 9.63 | 1 | 0.92 | 1 | 0.14 | -0.83 | -2.833* | 5 | 1.31 | 4 | 1.26 | -0.75 | -4.762* |
| Hymenoptera | 1 | 0.06 | 0 | 0 | 0 | 0.00 | -1.00 | -0.244* | 0 | 0 | 0 | 0.00 | -1.00 | -0.438* |
| Nematocera | 2 | 0.12 | 2 | 1.83 | 1 | 0.22 | 0.89 | 5.438 | 5 | 1.31 | 4 | 2.60 | 0.86 | 7.447 |
| Sternorrhyncha | 0 | 0 | 0 | 0 | 0 | 0.00 | NA | NA | 1 | 0.26 | 1 | 0.03 | NA | NA |
| Trichoptera | 0 | 0 | 2 | 1.83 | 1 | 0.22 | NA | NA | 6 | 1.57 | 4 | 0.82 | NA | NA |
| Amphipoda | 496 | 28.79 | 3 | 2.75 | 3 | 1.08 | -0.86 | -4.894* | 27 | 7.09 | 11 | 30.30 | -0.64 | -6.995* |
| *Daphnia* | 1 | 0.06 | 2 | 1.83 | 1 | 0.22 | 0.94 | 7.936 | 27 | 7.09 | 7 | 7.02 | 0.99 | 61.163* |
| Ostracoda | 0 | 0 | 0 | 0 | 0 | 0.00 | NA | NA | 21 | 5.51 | 9 | 4.88 | NA | NA |
| Acari | 11 | 0.64 | 0 | 0 | 0 | 0.00 | -1.00 | -0.811* | 2 | 0.52 | 1 | 0.08 | -0.03 | -0.078 |
| Aranae | 0 | 0 | 0 | 0 | 0 | 0.00 | NA | NA | 1 | 0.26 | 1 | 0.03 | NA | NA |
| Pseudoscorpiones | 1 | 0.06 | 0 | 0 | 0 | 0.00 | -1.00 | -0.244* | 0 | 0 | 0 | 0.00 | -1.00 | -0.438* |
| Annelida | 0 | 0 | 0 | 0 | 0 | 0.00 | NA | NA | 1 | 0.26 | 1 | 2.31 | NA | NA |
| *Cacosternum* | 0 | 0 | 1 | 0.92 | 1 | 2.67 | NA | NA | 1 | 0.26 | 1 | 0.03 | NA | NA |
| Anuran egg | 1 | 0.06 | 73 | 66.97 | 3 | 25.03 | 1.00 | 298.326* | 169 | 44.36 | 6 | 34.68 | 1.00 | 385.142* |
| Tadpole spec | 81 | 4.7 | 12 | 11.01 | 10 | 100.00 | 0.46 | 3.253 | 10 | 2.62 | 10 | 2.51 | -0.23 | -1.410 |
| *Xenopus* tadpole | 694 | 40.28 | 0 | 0 | 0 | 0.00 | -1.00 | -6.441* | 1 | 0.26 | 1 | 0.36 | -0.99 | -11.460* |
| N, total number of individuals obtained; N% percentage of N; Freq, frequency of occurrence; Freq % percentages of frequency of occurrence; *IRI* (%), index of relative importance; *E**, Electivity index; *χ^2^* = Chi-square residuals, significant values are marked with an asterisk. | | | | | | | | | | | | | | |

**Table S5:** Prey categories consumed by *Xenopus laevis*, *Xenopus gilli* and obtained during habitat sampling at the Kleinmond for large frogs.

| **Kleinmond**  **52 – 72 mm** | **Environment** | | ***Xenopus laevis*** n = 33 | | | | | | |  | ***Xenopus gilli*** n = 60 | | | | |  |
| --- | --- | --- | --- | --- | --- | --- | --- | --- | --- | --- | --- | --- | --- | --- | --- | --- |
| **Order** | Ne | Ne (%) | N | N (%) | Freq | *IRI* (%) | *E** | *χ^2^* | | | N | N (%) | Freq | *IRI* (%) | *E** | *χ^2^* |
| Coleoptera | 260 | 15.09 | 36 | 3.3 | 14 | 7.00 | -0.68 | | -9.953* | | 38 | 7.47 | 18 | 6.90 | -0.36 | -4.206* |
| Ephemeroptera | 9 | 0.52 | 0 | 0 | 0 | 0.00 | -1.00 | | -2.376* | | 0 | 0 | 0 | 0.00 | -1.00 | -1.603* |
| Heteroptera | 166 | 9.63 | 17 | 1.56 | 7 | 2.55 | -0.74 | | -8.539* | | 7 | 1.38 | 3 | 0.14 | -0.76 | -5.868* |
| Hymenoptera | 1 | 0.06 | 0 | 0 | 0 | 0.00 | -1.00 | | -0.792* | | 0 | 0 | 0 | 0.00 | -1.00 | -0.534* |
| Nematocera | 2 | 0.12 | 5 | 0.46 | 3 | 0.16 | 0.60 | | 3.343 | | 9 | 1.77 | 6 | 0.30 | 0.88 | 11.154 |
| Trichoptera | 0 | 0 | 1 | 0.09 | 1 | 0.01 | NA | | NA | | 0 | 0 | 0 | 0.00 | NA | NA |
| Zygoptera | 0 | 0 | 1 | 0.09 | 1 | 0.04 | NA | | NA | | 0 | 0 | 0 | 0.00 | NA | NA |
| Amphipoda | 496 | 28.79 | 3 | 0.27 | 3 | 0.10 | -0.99 | | -17.470* | | 5 | 0.98 | 2 | 0.13 | -0.95 | -11.481* |
| *Daphnia* | 1 | 0.06 | 434 | 39.74 | 1 | 5.55 | 1.00 | | 547.131* | | 208 | 40.86 | 8 | 10.27 | 1.00 | 388.711* |
| Ostracoda | 0 | 0 | 1 | 0.09 | 1 | 0.01 | NA | | NA | | 2 | 0.39 | 1 | 0.01 | NA | NA |
| Acari | 11 | 0.64 | 1 | 0.09 | 1 | 0.01 | -0.75 | | -2.246* | | 2 | 0.39 | 2 | 0.02 | -0.22 | -0.644 |
| Pseudoscorpiones | 1 | 0.06 | 0 | 0 | 0 | 0.00 | -1.00 | | -0.792* | | 0 | 0 | 0 | 0.00 | -1.00 | -0.534* |
| *Cacosternum* | 0 | 0 | 0 | 0 | 0 | 0.00 | NA | | NA | | 2 | 0.39 | 2 | 0.02 | NA | NA |
| Anuran egg | 1 | 0.06 | 565 | 51.74 | 10 | 67.03 | 1.00 | | 712.518* | | 172 | 33.79 | 7 | 7.26 | 1.00 | 321.342* |
| Tadpole spec | 81 | 4.7 | 20 | 1.83 | 16 | 100.00 | -0.45 | | -4.323* | | 48 | 9.43 | 38 | 100.00 | 0.37 | 5.171* |
| *Xenopus* tadpole | 694 | 40.28 | 0 | 0 | 0 | 0.00 | -1.00 | | -20.867* | | 3 | 0.59 | 2 | 0.03 | -0.98 | -13.864* |
| N, total number of individuals obtained; N% percentage of N; Freq, frequency of occurrence; Freq % percentages of frequency of occurrence; *IRI* (%), index of relative importance; *E**, Electivity index; *χ^2^* = Chi-square residuals, significant values are marked with an asterisk. | | | | | | | | | | | | | | | | |

**Table S6:** Prey categories consumed by *Xenopus laevis* and obtained during habitat sampling at the Kleinmond for very large frogs.

| **Kleinmond**  **>72 mm** | **Environment** | | ***Xenopus laevis*** n = 33 | | | | |  |
| --- | --- | --- | --- | --- | --- | --- | --- | --- |
| **Order** | Ne | Ne (%) | N | N (%) | Freq | *IRI* (%) | *E** | *χ^2^* |
| Coleoptera | 260 | 15.09 | 19 | 3.86 | 3 | 34.03 | -0.62 | -6.289* |
| Ephemeroptera | 9 | 0.52 | 0 | 0 | 2 | 0.00 | -1.00 | -1.585* |
| Heteroptera | 166 | 9.63 | 4 | 0.81 | 3 | 0.99 | -0.85 | -6.220* |
| Hymenoptera | 1 | 0.06 | 0 | 0 | 1 | 0.00 | -1.00 | -0.528* |
| Nematocera | 2 | 0.12 | 2 | 0.41 | 1 | 0.15 | 0.56 | 1.929 |
| Amphipoda | 496 | 28.79 | 0 | 0 | 3 | 0.00 | -1.00 | -11.767* |
| *Daphnia* | 1 | 0.06 | 4 | 0.81 | 1 | 0.31 | 0.87 | 7.042 |
| Acari | 11 | 0.64 | 0 | 0 | 2 | 0.00 | -1.00 | -1.752* |
| Pseudoscorpiones | 1 | 0.06 | 0 | 0 | 1 | 0.00 | -1.00 | -0.528* |
| *Cacosternum* | 0 | 0 | 2 | 0.41 | 0 | 12.90 |  | NA |
| Anuran egg | 1 | 0.06 | 399 | 81.1 | 1 | 100.00 | 1.00 | 754.639* |
| Tadpole spec | 81 | 4.7 | 46 | 9.35 | 2 | 41.00 | 0.36 | 4.918* |
| *Xenopus* tadpole | 694 | 40.28 | 7 | 1.42 | 3 | 15.97 | -0.96 | -13.416* |
| N, total number of individuals obtained; N% percentage of N; Freq, frequency of occurrence; Freq % percentages of frequency of occurrence; *IRI* (%), index of relative importance; *E**, Electivity index; *χ^2^* = Chi-square residuals, significant values are marked with an asterisk. | | | | | | | | |
